# Seed layer formation by deposition of microcrystallites on a revolving substrate: modeling of the effective linear elastic, piezoelectric, and dielectric coefficients

**DOI:** 10.1107/S2052520624010436

**Published:** 2024-11-21

**Authors:** Arthur Ballato, John Ballato

**Affiliations:** a Holcombe Department of Electrical and Computer Engineering Clemson University Clemson SC29634 USA; b Department of Materials Science and Engineering Clemson University Clemson SC29634 USA; University of Geneva, Switzerland

**Keywords:** deposition, sputtering, rotating substrate, crystallites, dielectric permittivity, piezoelectricity, elasticity, zinc oxide, engineered polycrystals, seed layers, texture

## Abstract

Averaging of material coefficients of crystallites deposited at an angle to a rotating substrate is considered. A simple model is proposed, and applied to determine effective linear dielectric, piezoelectric, and elastic constants of all Laue groups. While these represent tensors of rank 2, 3, and 4, the method applies generally to tensors of any rank. Results are then particularized for 6*mm* point symmetry crystals, and applied numerically to zinc oxide, ZnO. It is shown that, by means of the rotating substrate method, depositions may be achieved having the equivalent of hexagonal anisotropy, enabling the creation of ‘engineered’ structures.

## Abstract 2

The rotating-substrate method of crystallite deposition is modeled, allowing computation of effective material coefficients of the layers resulting from the averaging. A numerical example particularized to ZnO is provided.

## 1. Introduction

Deposition technology is critical in micro- and nano-electronic fabrication of sensors, transducers, filters, energy harvesters, resonators, *etc*. (Martin, 2010 ▸; Behrisch, 2013*a* ▸, Behrisch, 2013*b* ▸; Bundesmann & Neumann, 2018 ▸). These devices most often consist of multi-layer structures, the reliability and functionality of which depend on the ability to create uniform and stable layer interfaces. One modality for assuring interface compatibility is an initial deposition between working layers of a thin ‘seed’ or buffer layer, whose material and composition are chosen to afford compatible adhesion and to provide suitable conditions for subsequent uniform working layer growth (Clement *et al.*, 2012 ▸; Noh *et al.*, 2014 ▸; Li *et al.*, 2020 ▸). This is particularly important when forming structures comprised of single, or quasi-single crystal piezoelectric working layers. The axial orientation of these, with respect to the substrate normal, *i.e.* the texture (Bunge, 1982 ▸; Suwas & Gurao, 2008 ▸) allows their effective piezo-coupling and elastic values to be optimized (Bjurström *et al.*, 2006 ▸; Yanagitani *et al.*, 2007*a* ▸; Du *et al.*, 2009 ▸; Moreria *et al.*, 2011 ▸).

It is found that often superior seed layers are achieved by deposition of microcrystallites at a single angle to a revolving substrate (Stedile *et al.*, 1994 ▸; Auger *et al.*, 2002 ▸; Mertin *et al.*, 2018 ▸). The substrate rotation averages the crystallite properties, resulting in a polycrystalline aggregate film with material properties modified from the single crystal values of the seed material. After deposition of the seed layer, the substrate rotation is halted, and deposition of the textured film is commenced. This oriented film may be of the same material as that of the seed, or may, indeed, be of any material, deposited at the same angle as that of the seed microcrystals, or at any desired angle. This is arranged by suitable adjustment of the deposition apparatus parameters.

This work derives effective values for the elastic stiffnesses, piezoelectric stress coefficients, and dielectric permittivities of the seed layer using a simple model outlined below. The model may easily be expanded to accommodate a more realistic orientation distribution function characterizing the incident seed layer flux, rather than a fixed single deposition angle. It is noted that material values deviate from those of the bulk as crystallite size diminishes; this is usual when the micro/nanoboundary is crossed. The effective crystallite material coefficients are to be substituted for those of the bulk, with no changes in the averaging procedure described herein.

If the seed layer is not constrained to be ‘thin’, then other potential configurations emerge. It becomes possible to construct an assortment of tailored structures such as acoustic and optical Bragg reflectors consisting of alternating high- and low-impedance layers (Newell, 1964 ▸; Link *et al.*, 2006 ▸; Dvoesherstov *et al.*, 2013 ▸), as utilized, for example, in solidly mounted resonators (Lakin, 2003 ▸; Iborra *et al.*, 2012 ▸; Nguyen *et al.*, 2019 ▸). Other options are enabled as well, such as superlattices (Schuller, 1980 ▸; Akçakaya *et al.*, 1990 ▸; Bykhovski *et al.*, 1997 ▸), including Fibonacci and Moiré designs (MacDonald, 1988 ▸; Dean *et al.*, 2013 ▸; Mouldi & Kanzari, 2013 ▸). A simple example is offered in Section 4 where ZnO microcrystals are considered to be deposited with (103) planes parallel to the revolving substrate plane. Substrate rotation, and consequent angular averaging (see Section 2), produces an engineered crystal, again with 6*mm* symmetry, albeit with modified material coefficients, which are computed.

## 2. The model

**Figure 1:**
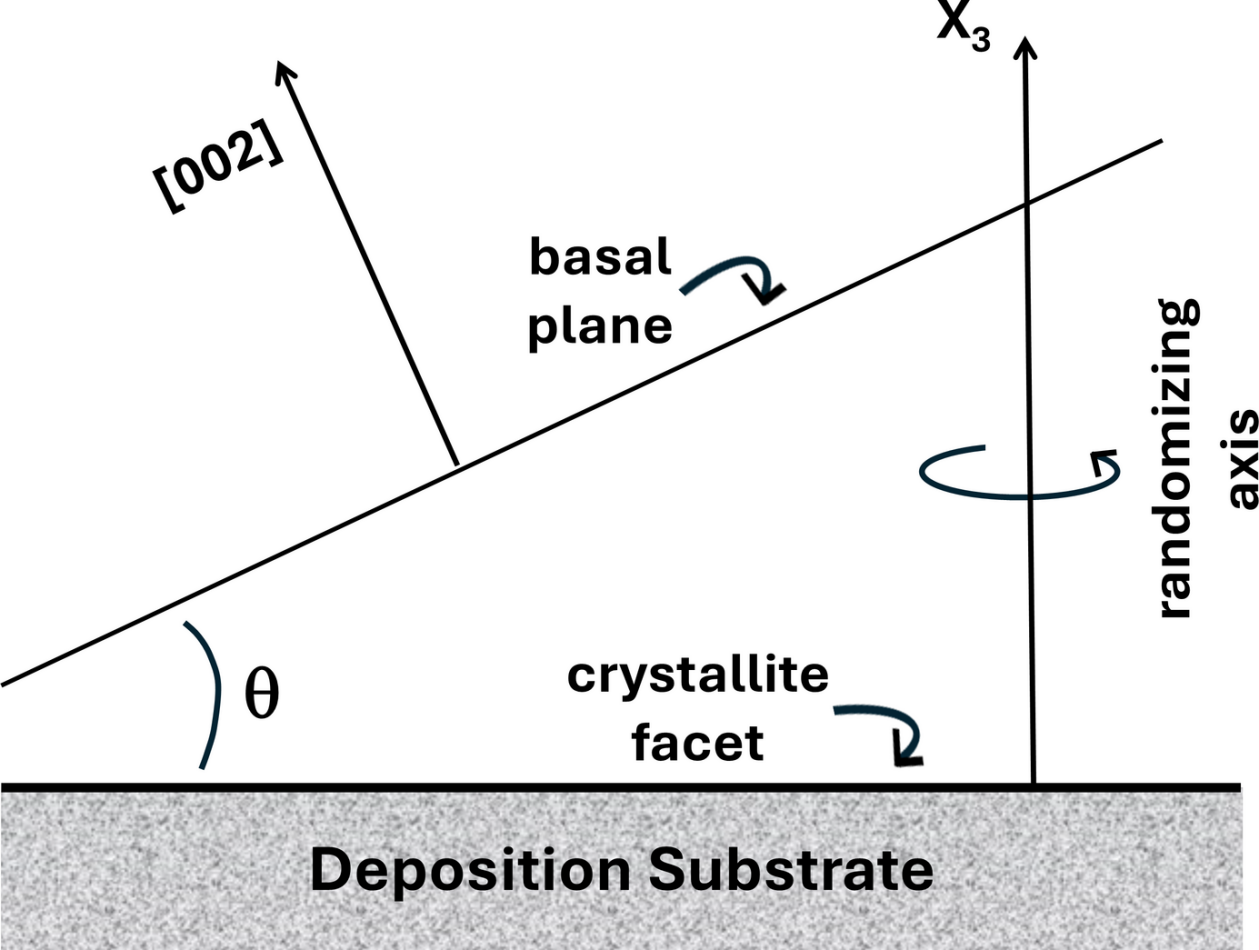
Schematic of the deposition geometry. The crystallite facet lies upon the substrate; its *x*_3_ axis, shown as [002], subtends cone angle θ with respect to the substrate randomizing axis *X*_3_.

Microcrystallites are deposited on a revolving substrate, all with a specified facet lying in the substrate plane. The *x*_3_ axis of the crystallite is thus inclined with respect to the substrate normal (*X*_3_) by a facet angle θ; see Fig. 1 ▸. The substrate revolution, in angle φ, creates a distribution of crystallite normals ([002] in the diagram) that form a cone about the substrate normal. The model assumes a uniform areal distribution of crystallites on the substrate. A suitable angular averaging then yields effective values 〈*q*〉 = 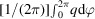, where *q* is the quantity to be averaged. In the following sections this averaging is carried out schematically on the point-group matrices characterizing the linear elastic, piezo, and dielectric crystal properties. These three families of phenomenological coefficients are much utilized in electro-acoustic applications and are used here to illustrate the results applied to tensors of ranks 2, 3, and 4 in their matrix form. The model may be extended to material tensors of arbitrary rank.

Axial conventions, and associated nomenclature, for the point groups are given, *e.g.* in Institute of Radio Engineers Standards Committee (1949 ▸); symmetry relations relating the tensorial material coefficients are succinctly discussed by Bhagavantam & Suryanarayana (1949 ▸), Fumi (1952*a* ▸), Fumi (1952*b* ▸), Fumi (1952*c* ▸), Fieschi (1957 ▸), and in engineering format by Cady (1946 ▸), Cady (1964 ▸) Mason (1966 ▸), Nye (1985 ▸). Tensor coefficients describing the elastic, piezo, and dielectric effects, are transformed according to standard rules (Bond, 1943 ▸; Juretschke, 1952 ▸; Bechmann, 1960 ▸; Hearmon, 1957 ▸). Initially, the crystallite *x*_3_ axis is normal to the substrate reference plane, and a first rotation about its *x*_1_ or *x*_2_ axis carries it to the configuration shown in Fig. 1 ▸. The elastic, piezoelectric, and dielectric matrices resulting from this first rotation are denoted [*c*′], [*e*′], and [ɛ′], respectively. Subsequent rotation of the crystallite sitting on the substrate plane is described by a second rotation about its *x*_3_′ axis; the averaging is carried out on the resulting transformed quantities, with final averaged results denoted as 〈*c*″〉, 〈*e*″〉, and 〈ɛ″〉.

## 3. Model results for all Laue groups

In this section are given the averaged components, 〈″〉, of the dielectric, piezoelectric, and elastic tensors for all Laue groups (Bechmann & Hearmon, 1969 ▸; Brendel, 1979 ▸).

### 3.1. Linear dielectric permittivities

Five distinct, symmetric, permittivity matrices, [ɛ], corresponding to Laue groups I, II, III, IV–VI, and VII, characterize the linear dielectric behavior of crystals (Cady, 1946 ▸, 1964 ▸; Mason, 1966 ▸; Nye, 1985 ▸). The geometry of Fig. 1 ▸ is realized by a rotation about an in-plane *x*_1_ or *x*_2_ axis by facet angle θ; this transforms these to five distinct matrices [ɛ′] in standard fashion. Anticipating the final result, these [ɛ′] matrices are not given explicitly. A further rotation about the resulting *x*_3_′ axis (normal to the substrate plane), and subsequent averaging by the revolving substrate produces two distinct 〈ɛ″〉 matrices, the components of which are given in terms of the components of [ɛ′] in Table 1 ▸, with 〈ɛ_22_″〉 = 〈ɛ_11_″〉, and zero off-diagonal elements. In terms of the components of the original [ɛ] matrices, the final result is given in Table 2 ▸.

### 3.2. Linear piezoelectric stress moduli

Sixteen distinct matrices [*e*], characterize the linear piezoelectric behavior of the 20 piezoelectric classes (Cady, 1946 ▸, 1964 ▸; Mason, 1966 ▸; Nye, 1985 ▸). Each of the Laue groups is represented. The pairs (4, 6), (422, 622), (4*mm*, 6*mm*), and (23, 43*m*) share the same matrices. The geometry of Fig. 1 ▸ is realized by a rotation about an in-plane *x*_1_ or *x*_2_ axis by facet angle θ; this transforms each of these sixteen to one of five distinct matrices [*e*′] in standard fashion. The Laue group I results for both *x*_1_ and *x*_2_ rotations are identical, so there are but nine distinct [*e*′] matrices. Again, anticipating the final result, these [*e*′] matrices are not given explicitly. A further rotation about the resulting *x*_3_′ axis (normal to the substrate plane), and subsequent averaging by the revolving substrate produces three distinct 〈*e*″〉 matrices, that we denote as 〈*A*〉, 〈*B*〉, and 〈*C*〉. The structure of matrix 〈*A*〉 is given in Table 3 ▸, where the 〈*e*″〉 components are expressed in terms of the [*e*′] components; it is identical in form to the matrix of the unrotated classes (4, 6). Matrix 〈*B*〉 is that of matrix 〈*A*〉 with *e*_14_″ = *e*_25_″ = 0 and has the same structure as the matrix of the unrotated classes (4*mm*, 6*mm*). Finally, matrix 〈*C*〉 is that of matrix 〈*A*〉 with *e*_15_″ = *e*_24_″ = *e*_31_″ = *e*_32_″ = *e*_33_″ = 0 and has the same structure as the matrix of the unrotated classes (422, 622). The averaged results for the twenty piezoelectric classes are given in Table 4 ▸.

The pertinent [*e*′] matrix components are given explicitly in terms of the components of the original [*e*] matrices in Table 5 ▸ and Table 6 ▸ for Laue group I. These relations are easily specialized for the remaining Laue groups of higher symmetry.

### 3.3. Linear elastic stiffnesses

Nine distinct, symmetric, stiffness matrices, [*c*], corresponding to Laue groups I, II, III, IVa, IVb, Va, Vb, VI, and VII, characterize the linear elastic behavior of crystals (Cady, 1946 ▸; 1964 ▸; Mason, 1966 ▸; Nye, 1985 ▸). The geometry of Fig. 1 ▸ is realized by a rotation about an in-plane *x*_1_ or *x*_2_ axis by facet angle θ; this transforms each of these nine to matrices [*c*′] in standard fashion. A further rotation about the resulting *x*_3_′ axis, (normal to the substrate plane), and subsequent averaging by the revolving substrate produces, for all Laue groups, a single 〈*c*″〉 matrix with hexagonal symmetry. The components of 〈*c*″〉 are given in terms of the components of [*c*′] in Table 7 ▸. The pertinent [*c*′] matrix components are given explicitly in terms of the components of the original [*c*] matrices in Tables 8 ▸ and 9 ▸ for Laue group I. These relations are easily specialized for the remaining Laue groups of higher symmetry.

## 4. Example: zinc oxide, Laue group VIb, class 6*mm*

Zinc oxide, in its 6*mm* polytype, is similar in many ways to the III-nitrides, and to polarized ceramics. Its high piezoelectric coupling has led to its use in a wide variety of acousto-electronic devices. Provided here are the effective material coefficients, and piezoelectric coupling factors, of deposited ZnO crystallites subjected to the averaging procedure described in Section 2. The 6*mm* symmetry requires isotropy in the basal plane, hence all rotations (tilts) about any axis, (*e.g.x*_1_ or *x*_2_), in the (001) plane will yield identical results. We take the first rotation about *x*_1_ by a facet angle θ corresponding to the (103) plane (Kushibiki *et al.*, 2009 ▸); in the coordinate system of the substrate, this results in the equivalent of monoclinic symmetry. The averaging process then yields 〈″〉 quantities but again with 6*mm* symmetry. The numerical input values are taken from Jaffe & Berlincourt (1965 ▸) and Pearton *et al.* (2005 ▸).

### 4.1. Dielectric permittivities

A first rotation, about x_1_, results in [ɛ′] quantities as follows: ɛ_11_′ = ɛ_11_; ɛ_12_′ = ɛ_13_′ = 0; ɛ_22_′ = ɛ_11_*C*^2^ + ɛ_33_*S*^2^; 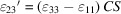; and ɛ_33_′ = ɛ_33_*C*^2^ + ɛ_11_*S*^2^. The resulting 〈ɛ″〉 components, taken from Table 2 ▸, and numerical values for ZnO referred to the facet plane (103), are given in Table 10 ▸.

### 4.2. Piezoelectric stress coefficients

A first rotation, about *x*_1_, results in [*e*′] quantities as follows: *e*_15_′ = *e*_15_*C*; *e*_16_′ = *e*_15_*S*; *e*_21_′ = *e*_31_*S*; *e*_22_′ = [*e*_33_*S*^2^ + (*e*_31_ + 2*e*_15_)*C*^2^]*S*; *e*_23_′ = [*e*_31_*S*^2^ + (*e*_33_ − 2*e*_15_)*C*^2^]*S*; *e*_24_′ = [*e*_33_*S*^2^ − *e*_31_*S*^2^ + *e*_15_(*C*^2^ − *S*^2^)]*C*; *e*_31_′ = *e*_31_*C*; *e*_32_′ = [*e*_31_*C*^2^ − 2*e*_15_*S*^2^ + *e*_33_*S*^2^]*C*; *e*_33_′ = [*e*_33_*C*^2^ + 2*e*_15_*S*^2^ + *e*_31_*S*^2^]*C*; and *e*_34_′ = [*e*_33_*C*^2^ − *e*_31_*C*^2^ − *e*_15_(*C*^2^ − *S*^2^)]*S*. The subsequent averaging, from Table 4 ▸, results in matrix 〈*B*〉 symmetry, with components as follows: 〈*e*_15_″〉 = 〈*e*_24_″〉 = (1/2)(*e*_15_′ + *e*_24_′) = (1/2)[2*e*_15_*C*^2^ + (*e*_33_− *e*_31_)*S*^2^]*C*; 〈*e*_31_″〉 = 〈*e*_32_″〉 = (1/2)(*e*_31_′ + *e*_32_′) = (1/2)[*e*_31_(1 + *C*^2^) + (*e*_33_ − 2*e*_15_)*S*^2^]*C*; 〈*e*_33_″〉 = [*e*_33_*C*^2^ + (*e*_33_ + 2*e*_15_)*S*^2^]*C*; numerical results are given in Table 11 ▸.

### 4.3. Elastic stiffnesses components

A first rotation, about *x*_1_, results in the [*c*′] quantities given in Table 12 ▸. When these are put into the 〈*c*″〉 matrix of Table 7 ▸, the entries of Table 13 ▸ result; numerical values for ZnO referred to the facet plane (103), are also given.

### 4.4. Computation of piezo-coupling values

Piezo-coupling coefficients determine efficacy of piezoelectric transduction. As measures thereof, ‘they appear in considerations of bandwidth and insertion loss in transducers and signal processing devices, in the location and spacing of critical frequencies of resonators, and in electrical/mechanical energy conversion efficiency in actuators and energy harvesters’ (Ballato & Ballato, 2023 ▸). Calculation of these quantities for the one-dimensional case of thickness vibrations of plates and films requires knowledge only of the effective dielectric, piezoelectric, and elastic tensors referred to the plate/film coordinates; these are the 〈″〉 quantities in our situation. An eigenvalue equation is solved, yielding three modal elastic stiffnesses, *c*_*m*_, three modal piezoelectric coefficients, *e*_*m*_, and the effective permittivity in the thickness direction, ɛ_3_. Then the coupling coefficients are given in the generic form |*k*_*m*_| = |*e*_*m*_|/(*c*_*m*_ɛ_3_)^1/2^ (Foster *et al.*, 1968 ▸; Wittstruck *et al.*, 2005 ▸; Ballato & Ballato, 2023 ▸).

**Figure 2:**
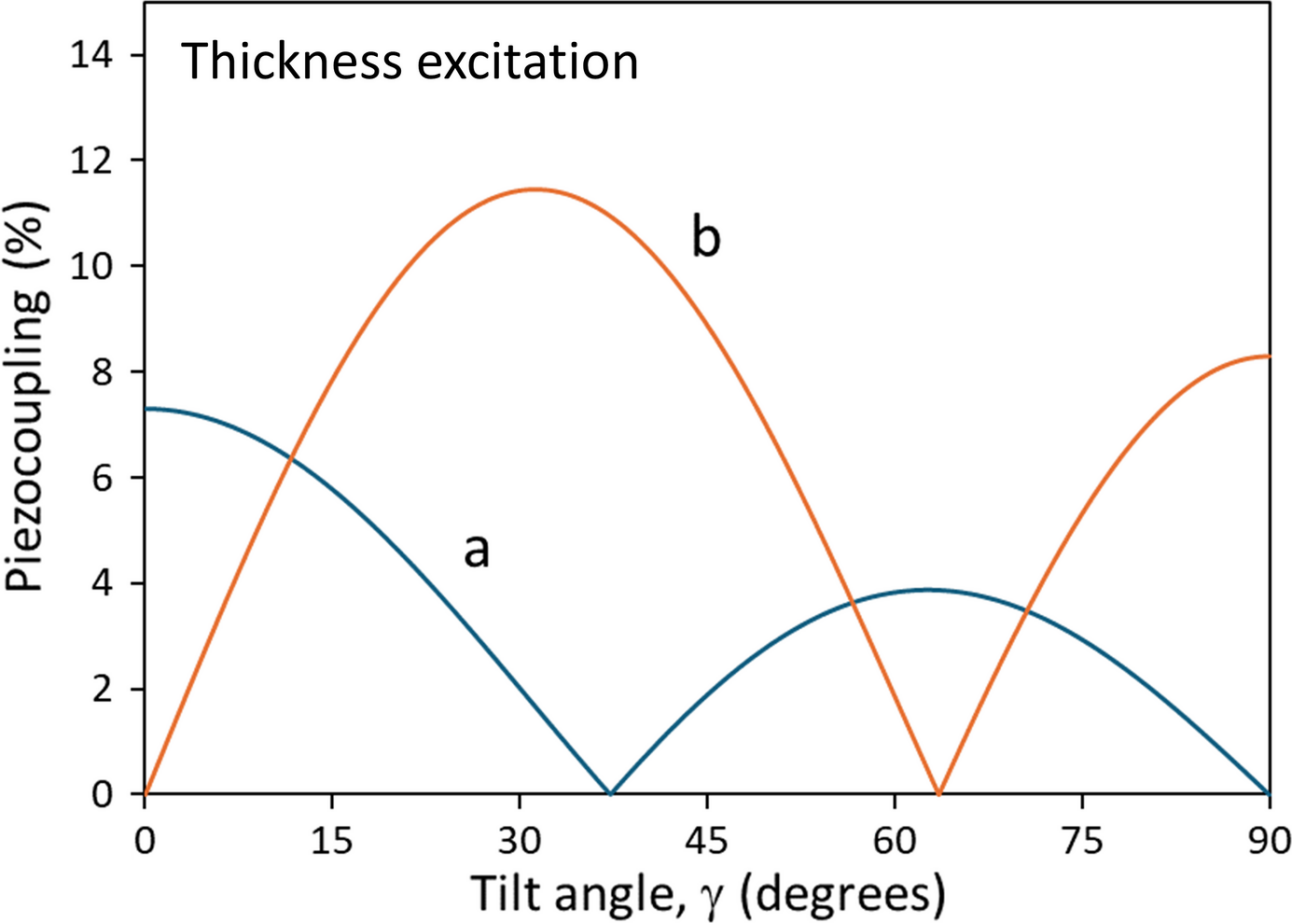
Piezo-couplings |*k*_*a*_| and |*k*_*b*_| of an engineered ZnO plate formed by averaging over facet angle θ ≃ 31.664°, plotted versus tilt (texture) angle γ. Driving electric field is normal to the substrate plane. Waves *a* and *b* are quasi-thickness extension, and quasi-thickness shear, respectively. Note the similar, but reduced, values of coupling compared with Foster *et al.* (1968 ▸), where the facet angle θ = 0°. Wave *c*, (pure shear) is inert to thickness-directed fields. In devices using the quasi-shear mode, a practical tilt angle would be at, or near, the value where |*k*_*a*_| = 0; here the *b* wave coupling is nearly a maximum.

In Table 14 ▸ are given the relevant data for averaged ZnO with zero facet angle. Corresponding wave velocities are *V*_*m*_ = (*c*_*m*_/ρ)^1/2^. The mass density of bulk crystalline ZnO is ρ ≃ 5.606 × 10^3^ (kg m^−3^) (Pearton *et al.*, 2005 ▸). Table 15 ▸ provides this data for averaged ZnO with facet angle of the (103) plane. The |*k*_*m*_| values are plotted in Fig. 2 ▸, as a function of tilt angle γ, for averaged ZnO with facet angle of the (103) plane.

## 5. Conclusions

The rotating substrate method of crystallite deposition is modeled, allowing computation of effective material coefficients of the layers resulting from the averaging. Modeling of the averaging applies generally to tensors of any rank, but is applied here to dielectric, piezoelectric, and elastic coefficients. A worked numerical example particularized to 6*mm* ZnO is provided. Layers comprised of crystallites deposited with zero facet angle are contrasted with those deposited on facet plane (103). It is proposed that the method enables creation of ‘engineered’ layered structures.

## Figures and Tables

**Table 1:** Averaged dielectric permittivities, 〈ɛ_*kk*_″〉, in terms of the singly rotated quantities, ɛ_*jj*_′

〈ɛ_*kk*_″〉	Groups I–VI	Group VII
〈ɛ_11_″〉	½(ɛ_11_′ + ɛ_22_′)	ɛ_11_′
〈ɛ_33_″〉	ɛ_33_′	ɛ_11_′

**Table 2:** Averaged dielectric permittivities for all Laue groups, for first rotation x_1_ or x_2_ C and S are cos(θ) and sin(θ), respectively, where θ is the angle of first rotation, the facet angle.

Rotation	Laue →	Group I	Group II	Group III	Groups IV–VI	Group VII
*x*_1_	〈ɛ_11_″〉	½(ɛ_11_ + ɛ_22_*C*^2^ + 2ɛ_23_*CS* + ɛ_33_*S*^2^)	½(ɛ_11_ + ɛ_22_*C*^2^ + ɛ_33_*S*^2^)		½(ɛ_11_ + ɛ_11_*C*^2^ + ɛ_33_*S*^2^)	ɛ_11_
	〈ɛ_33_″〉	ɛ_33_*C*^2^ − 2ɛ_23_*CS* + ɛ_22_*S*^2^	ɛ_33_*C*^2^ + ɛ_22_*S*^2^		ɛ_33_*C*^2^ + ɛ_22_*S*^2^	ɛ_11_
*x*_2_	〈ɛ_11_″〉	½(ɛ_22_ + ɛ_11_*C*^2^ − 2ɛ_13_*CS* + ɛ_33_*S*^2^)		½(ɛ_22_ + ɛ_11_*C*^2^ + ɛ_33_*S*^2^)	½(ɛ_11_ + ɛ_11_*C*^2^ + ɛ_33_*S*^2^)	ɛ_11_
	〈ɛ_33_″〉	ɛ_33_*C*^2^ + 2ɛ_13_*CS* + ɛ_11_*S*^2^		ɛ_33_*C*^2^ + ɛ_11_*S*^2^		ɛ_11_

**Table 3:** The 〈*e*″〉 components of matrix 〈*A*〉 expressed in terms of the singly rotated *e*′ elements

0	0	0	〈*e*_14_″〉	〈*e*_15_″〉	0	=	0	0	0	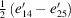	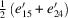	0
0	0	0	〈*e*_15_″〉	−〈*e*_14_″〉	0		0	0	0	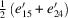	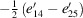	0
〈*e*_31_″〉	〈*e*_31_″〉	〈*e*_33_″〉	0	0	0		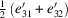	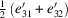	*e*_33_″	0	0	0

**Table 4:** Resulting matrix after second rotation about *x*_3_, and subsequent averaging by the revolving substrate

Laue →	I	II		III		IVa and VIa	IVa	IVb and VIb		IVb	Va	Vb		VIa	VIb	VIIa and VIIb
H-M →	1	2	*m*	222	*mm*2	4, 6	4	422, 622	4*mm*, 6*mm*	42*m*	3	32	3*m*	6	6*m*2	23, 43*m*
Rotation																
*x*_1_	〈*A*〉	〈*A*〉	〈*A*〉	〈*C*〉	〈*B*〉	〈*A*〉	〈*A*〉	〈*C*〉	〈*B*〉	〈*C*〉	〈*A*〉	〈*C*〉	〈*B*〉	〈*A*〉	〈*C*〉	〈*C*〉
*x*_2_	〈*A*〉	〈*C*〉	〈*B*〉	〈*C*〉	〈*B*〉	〈*A*〉	〈*A*〉	〈*C*〉	〈*B*〉	〈*C*〉	〈*A*〉	〈*A*〉	〈*A*〉	〈*A*〉	〈*B*〉	〈*C*〉

**Table 5:** Singly rotated *e*′ coefficients for triclinic Laue group I in terms of the unrotated *e* coefficients With θ the angle of first rotation about *x*_1_, *C* and *S* stand for cos(θ) and sin(θ), where θ is the facet angle.

*e*_14_′	=	*e*_14_(*C*^2^ − *S*^2^) + (*e*_13_ − *e*_12_)*CS*
*e*_15_′		*e*_15_*C*^2^ − *e*_16_*S*
*e*_24_′		(*e*_24_*C* + *e*_34_*S*)(*C*^2^ − *S*^2^) − [(*e*_22_*C* + *e*_32_*S*) − (*e*_23_*C* + *e*_33_*S*)]*CS*
*e*_25_′		(*e*_25_*C* + *e*_35_*S*)*C* − (*e*_26_*C* + *e*_36_*S*)*S*
*e*_31_′		*e*_31_*C* − *e*_21_*S*
*e*_32_′		(*e*_32_*C* − *e*_22_*S*)*C*^2^ + (*e*_33_*C* − *e*_23_*S*)*S*^2^ + 2(*e*_34_*C* − *e*_24_*S*)*CS*
*e*_33_′		(*e*_33_*C* − *e*_23_*S*)*C*^2^ + (*e*_32_*C* − *e*_22_*S*)*S*^2^ − 2(*e*_34_*C* − *e*_24_*S*)*CS*

**Table 6:** Singly rotated *e*′ coefficients for triclinic Laue group I in terms of the unrotated *e* coefficients With θ the angle of first rotation about *x*_2_, *C* and *S* stand for cos(θ) and sin(θ), where θ is the facet angle.

*e*_14_′	=	*e*_14_*C*^2^ − *e*_36_*S*^2^ + (*e*_16_ − *e*_34_)*CS*
*e*_15_′		(*e*_15_*C* − *e*_35_*S*)(*C*^2^ − *S*^2^) + [(*e*_11_ − *e*_13_)*C* + (*e*_33_ − *e*_31_)*S*]*CS*
*e*_24_′		*e*_24_*C* − *e*_26_*S*
*e*_25_′		*e*_25_(*C*^2^ − *S*^2^) + (*e*_21_ − *e*_23_)*CS*
*e*_31_′		(*e*_31_*C* + *e*_11_*S*)*C*^2^ + (*e*_33_*C* + *e*_13_*S*)*S*^2^ − 2(*e*_35_*C* + *e*_15_*S*)*CS*
*e*_32_′		*e*_32_*C* + *e*_12_*S*
*e*_33_′		(*e*_33_*C* + *e*_13_*S*)*C*^2^ + (*e*_31_*C* + *e*_11_*S*)*S*^2^ + 2(*e*_35_*C* + *e*_15_*S*)*CS*

**Table 7:** Averaged elastic stiffnesses for Laue groups I–VII as function of the *c*′ components The 〈**c**″〉 matrix possesses hexagonal symmetry.

〈*c*_λμ_″〉		*x*_1_ or *x*_2_ rotation
〈*c*_11_″〉	=	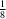 [3*c*_11_′ + 2*c*_12_′ + 3*c*_22_′ + 4*c*_66_′]
〈*c*_12_″〉		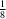 [*c*_11_′ + 6*c*_12_′ + *c*_22_′ − 4*c*_66_′]
〈*c*_13_″〉		½[*c*_13_′ + *c*_23_′]
〈*c*_33_″〉		*c*_33_′
〈*c*_44_″〉		½[*c*_44_′ + *c*_55_′]
〈*c*_66_″〉		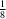 [*c*_11_′ − 2*c*_12_′ + *c*_22_′ + 4*c*_66_′]

**Table 8:** Transformed elastic stiffnesses for triclinic Laue group I, as function of the first rotation *x*_1_ Only the pertinent *c*′, needed for insertion into Table 7 ▸, are listed. *C* and *S* are cos(θ) and sin(θ), respectively, where θ is the facet angle.

*c*_11_′	=	*c*_11_
*c*_22_′		*c*_22_*C*^4^ + *c*_33_*S*^4^ + 2[2(*c*_24_*C*^2^ + *c*_34_*S*^2^) + (*c*_23_ + 2*c*_44_)*CS*]*CS*
*c*_33_′		*c*_33_*C*^4^ + *c*_22_*S*^4^ + 2[−2(*c*_34_*C*^2^ + *c*_24_*S*^2^) + (*c*_23_ + 2*c*_44_)*CS*]*CS*
*c*_44_′		*c*_44_(*C*^4^ + *S*^4^) + [2(*c*_34_ − *c*_24_)(*C*^2^ − *S*^2^) + (*c*_22_ + *c*_33_ − 2*c*_23_ − 2*c*_44_)*CS*]*CS*
*c*_55_′		*c*_55_*C*^2^ − 2*c*_56_*CS* + *c*_66_*S*^2^
*c*_66_′		*c*_66_*C*^2^ + 2*c*_56_*CS* + *c*_55_*S*^2^
*c*_12_′		*c*_12_*C*^2^ + 2*c*_14_*CS* + *c*_13_*S*^2^
*c*_13_′		*c*_13_*C*^2^ − 2*c*_14_*CS* + *c*_12_*S*^2^
*c*_23_′		*c*_23_(*C*^4^ + *S*^4^) + [2(*c*_34_ − *c*_24_)(*C*^2^ − *S*^2^) + (*c*_22_ + *c*_33_ − 4*c*_44_)*CS*]*CS*
(*c*_44_′ − *c*_23_′)		(*c*_44_ − *c*_23_)

**Table 9:** Transformed elastic stiffnesses for triclinic Laue group I, as function of the first rotation *x*_2_ Only the pertinent *c*′, needed for insertion into Table 7 ▸, are listed. *C* and *S* are cos(θ) and sin(θ), respectively, where θ is the facet angle.

*c*_11_′	=	*c*_11_*C*^4^ + *c*_33_*S*^4^ − 2[2(*c*_15_*C*^2^ + *c*_35_*S*^2^) − (*c*_13_ + 2*c*_55_)*CS*]*CS*
*c*_22_′		*c*_22_
*c*_33_′		*c*_33_*C*^4^ + *c*_11_*S*^4^ + 2[2(*c*_35_*C*^2^ + *c*_15_*S*^2^) + (*c*_13_ + 2*c*_55_)*CS*]*CS*
*c*_44_′		*c*_44_*C*^2^ + 2*c*_46_*CS* + *c*_66_*S*^2^
*c*_55_′		*c*_55_(*C*^4^ + *S*^4^) + [2(*c*_15_ − *c*_35_)(*C*^2^ − *S*^2^) + (*c*_11_ + *c*_33_ − 2*c*_13_ − 2*c*_55_)*CS*]*CS*
*c*_66_′		*c*_66_*C*^2^ − 2*c*_46_*CS* + *c*_44_*S*^2^
*c*_12_′		*c*_12_*C*^2^ − 2*c*_25_*CS* + *c*_23_*S*^2^
*c*_13_′		*c*_13_(*C*^4^ + *S*^4^) + [2(*c*_15_ − *c*_35_)(*C*^2^ − *S*^2^) + (*c*_11_ + *c*_33_ − 4*c*_55_)*CS*]*CS*
*c*_23_′		*c*_23_*C*^2^ + 2*c*_25_*CS* + *c*_12_*S*^2^
(*c*_55_′ − *c*_13_′)		(*c*_55_ − *c*_13_)

**Table 10:** Averaged permittivities (pF m^−1^) for class 6*mm*, particularized for ZnO with facet plane (103)

〈ɛ_11_″〉	½(ɛ_11_′ + ɛ_22_′)	=	½(ɛ_11_ + ɛ_11_*C*^2^ + ɛ_33_*S*^2^)	=	74.38
〈ɛ_33_″〉	ɛ_33_′		ɛ_33_*C*^2^ + ɛ_11_*S*^2^		77.03

**Table 11:** Averaged piezoelectric stress coefficients (C m^−2^) for class 6*mm*, particularized for ZnO with facet plane (103)

0	0	0	0	〈*e*_15_″〉	0	=	0	0	0	0	−0.155	0
0	0	0	〈*e*_15_″〉	0	0		0	0	0	−0.155	0	0
〈*e*_31_″〉	〈*e*_31_″〉	〈*e*_33_″〉	0	0	0		−0.185	−0.185	+0.289	0	0	0

**Table 12:** Resulting elastic stiffnesses from a first rotation of a 6*mm* crystal about its *x*_1_ axis. *C* and *S* are cos(θ) and sin(θ), respectively, where θ is the facet angle

*c*_11_′	=	*c*_11_
*c*_12_′		*c*_12_*C*^2^ + *c*_13_*S*^2^
*c*_13_′		*c*_13_*C*^2^ + *c*_12_*S*^2^
*c*_22_′		*c*_11_*C*^4^ + *c*_33_*S*^4^ + 2(*c*_13_ + 2*c*_44_)*C*^2^*S*^2^
*c*_23_′		*c*_13_(*C*^4^ + *S*^4^) + (*c*_11_ + *c*_33_ − 4*c*_44_)*C*^2^*S*^2^
*c*_33_′		*c*_33_*C*^4^ + *c*_11_*S*^4^ + 2(*c*_13_ + 2*c*_44_)*C*^2^*S*^2^
*c*_44_′		*c*_44_(*C*^4^ + *S*^4^) + (*c*_11_ + *c*_33_ − 2*c*_13_ − 2*c*_44_)*C*^2^*S*^2^
*c*_55_′		*c*_44_*C*^2^ + *c*_66_*S*^2^
*c*_66_′		*c*_66_*C*^2^ + *c*_44_*S*^2^
(*c*_44_′ − *c*_23_′)		(*c*_44_ − *c*_23_)

**Table 13:** Averaged elastic stiffness coefficients (GPa) for class 6*mm*, with numerical values particularized for ZnO with facet plane (103)

〈*c*_11_″〉	=	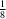 [*c*_11_(3 + 2*C*^2^) + 3(*c*_11_*C*^4^ + *c*_33_*S*^4^) + 2(*c*_13_ + 2*c*_44_)(1 + 3*C*^2^)*S*^2^]	=	205.72
〈*c*_12_″〉		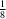 [*c*_11_(1 + 6*C*^2^) + (*c*_11_*C*^4^ + *c*_33_*S*^4^ − 4*c*_44_*S*^4^) − 16*c*_66_*C*^2^ + 2*c*_13_(3 + *C*^2^)*S*^2^]		117.34
〈*c*_13_″〉		½[*c*_13_*C*^2^ + *c*_13_(*C*^4^ + *S*^4^) + *c*_11_(1 + *C*^2^) + (*c*_33_*C*^2^ − 4*c*_44_*C*^2^ − 2*c*_66_)*S*^2^]		111.55
〈*c*_33_″〉		[*c*_33_*C*^4^ + *c*_11_*S*^4^ + 2(*c*_13_ + 2*c*_44_)*C*^2^*S*^2^]		202.66
〈*c*_44_″〉		½[*c*_44_*C*^2^ + *c*_44_(*C*^4^ + *S*^4^) + *c*_66_*S*^2^ + (*c*_11_ + *c*_33_ − 2*c*_13_ − 2*c*_44_)*C*^2^*S*^2^]		46.78
〈*c*_66_″〉		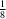 [8*c*_66_*C*^2^ + (*c*_11_ + *c*_33_ − 2*c*_13_)*S*^2^ + 4*c*_44_(1 + *C*^4^)*S*^2^] = (〈*c*_11_″〉 − 〈*c*_12_″〉)/2		44.19

**Table 14:** Effective elastic stiffnesses, *c*_*m*_, piezoelectric coupling coefficients, |*k*_*m*_|, and deviation angles, |δ_*a*_| of ZnO thin plates or films having tilt (texture) angles γ = 0°(15°)90° about an axis lying in the basal plane (001) Subscript *m* denotes the wave: *a* the quasi-longitudinal, and *b*, *c* the fast and slow quasi-shears, respectively. Piezo-coupling |*k*_*c*_| ≡ 0. Angle |δ_*a*_| gives the offset of the *a* wave motion from the wave progression direction, which is parallel to the plate/film thickness.

Quantity	Unit	γ = 0°	γ = 15°	γ = 30°	γ = 45°	γ = 60°	γ = 75°	γ = 90°
c_*a*_	GPa	228.07	220.11	205.33	201.12	206.64	209.91	210.00
c_*b*_		42.50	48.36	58.23	57.80	50.14	46.94	47.24
c_*c*_		42.50	42.62	42.95	43.40	43.85	44.18	44.30
|*k*_*a*_|	%	27.36	23.16	10.19	6.08	13.66	10.44	0
|*k*_*b*_|		0	25.82	37.47	29.93	5.81	20.62	31.66
|δ_*a*_|	°	0	3.74	3.28	0.66	1.86	0.74	0

**Table 15:** The same quantities defined in Table 14 ▸, but for ZnO microcrystallites that have undergone the averaging procedure described in the text (see Section 2) to create an engineered crystal with 6*mm* symmetry For the entries given, the facet angle for the averaging is that of the (103) plane, (θ ≃ 31.664°). The engineered crystal is then tilted by angles γ = 0°(15°)90° about an axis lying in its basal plane (001), *i.e.* the plane of the substrate, as a basis for comparison with the entries in Table 14 ▸, but more importantly, to suggest that untextured, engineered, crystals may be created to meet desired design criteria

Quantity	Unit	γ = 0°	γ = 15°	γ = 30°	γ = 45°	γ = 60°	γ = 75°	γ = 90°
c_*a*_	GPa	203.74	203.66	203.86	204.73	205.60	205.81	205.72
c_*b*_		46.78	46.96	47.05	46.69	46.45	46.80	47.11
c_*c*_		46.78	46.61	46.13	45.48	44.84	44.36	44.19
|*k*_*a*_|	%	7.29	5.76	2.01	1.89	3.82	2.93	0
|*k*_*b*_|		0	7.87	11.44	8.89	1.86	5.32	8.30
|δ_*a*_|	°	0	0.05	0.23	0.34	0.19	0.01	0
